# The Brooklyn Dental Association

**Published:** 1864-09

**Authors:** Wm. C. Horne


### Proceedings of Societies.

### THE BROOKLYN DENTAL ASSOCIATION.

At Dr. Hurd's Brooklyn E. D., May 25, 1864.

REPORTED BY DR. WM. C. HORNE.

A large part of the evening was consumed in the consideration of a remarkable case of alveolar abscess, brought up by Dr. Horne. Dr. John Allen read a paper detailing very graphically a case of excessive hemorrhage following the extraction of ten teeth and roots from the mouth of a female patient; with the means used for stopping the effusion which were ultimately successful. In his summing up Dr. Allen laid down the following rules: 1st. Arrive at a correct knowledge of the pathological condition of the patient before extracting so many teeth. 2d. Where a hemorrhagic diathesis is found to exist, not more than one or two teeth should be extracted. 3d. Where hemorrhage does occur, never cease your efforts till the effusion is stopped. Dr. Francis read a very good paper on the subject for the evening: "Temporary Fillings" which he was requested to contribute for publication. He detailed his method of procedure where time was brief; also when a nerve pulp was insufficiently protected. In such cases a plug of Hill's stopping, or Bevin's Filling was excellent as a temporary protector of the tooth. Dr. Wm. H. Atkinson said the perchlorid of iron is the Samson which can always be relied on in cases of hemorrhage. Pellets of cotton or, wedges of wood, firmly pressed into the sockets, are generally sufficient; perhaps better than either, would be to return the extracted fangs, (dipped in some styptic) to their places. He highly recommended the summing up of Dr. Allen's paper. In cases where a hemorrhagic diathesis is known or supposed to be present, it may be guarded against by the administration, some days in advance of the extraction, of muriated tincture of iron. When he had any need of assistance, he should prefer a dental friend to either physician or surgeon. Dr. J. Allen stated that in the present case, time was precious, and he called upon the first good man without regard to his profession. Dr. Atkinson was glad to hear Dr. Allen's explanation; he had taken the gentleman's measure long ago, and never knew him derelict to duty.

Dr. C. P. Fitch said the subject for the evening, "Temporary Fillings," had been ignored. He commended Dr. Francis' paper, and agreed with the conclusions and course of practice there laid down. He condemned *in toto* the use of plastic materials to cover up uncleanness and lack of ability; while there were occasions where temporary fillings were not only excusable, but necessary. To turn to the subject of hemorrhagic diathesis: Canada fleabane, (*Canadensis Erigerou,*) is one of the first hemostatics. Twenty or thirty drops administered at short intervals during as many minutes, will act with great promptness upon the column of the blood, by a succession of shocks. The plant must be gathered during the flowering season to secure this quality.

DR. JOHN ALLEN had used various styptics and generally found tannic acid fully sufficient; he prefers it to the persulphate of iron in all ordinary cases, because of the near approach to an escharotic of the latter agent, which is an objection to its use, he places great reliance on compresses.

DR. WM. B. HURD said the great point in his estimation was in forcing the cotton firmly into the socket; do it thoroughly, and there need be little fear but that the bleeding will cease.

DR. C. P. FITCH said that will do except where there is bleeding from all the abraded surfaces; as is the case when the blood is thin. A plasticity of the blood in such cases should be corrected; also the application of the actual or potential cantery to the mouths of the wounds might be efficacious.

DR. ROWE inquired whether hemorrhage from the sockets of the extracted teeth might not be stopped by stitching the gums together.

DR. ATKINSON said stitching the gums might answer if the blood flowed from the anterior portion of the mouth. Hemorrhagic diathesis is simply a condition in which the blood oozes from all the wounded surfaces. The case narrated was not such, but one of secondary hemorrhage; and this is just what we are to dread. Here it was induced by the patients' taking a warm bath. When a root is extracted, the blood vessel which enters the socket being attached to the bony wall can not contract; ordinarily coagulation stops the flow, but when this fails the connection may be broken up by forcing a burr to the bottom of the socket and rotating it a few times.

DR. JOHN ALLEN stated that the patient whose case he had detailed has long been subject to disease of the kidneys; this had induced a hemorrhagic diathesis, which he felt was the true explanation of the excessive bleeding.

[The subject of "Temporary Fillings" was ordered for the next meeting.]

